# Towards reproducible radiomics research: introduction of a database for radiomics studies

**DOI:** 10.1007/s00330-023-10095-3

**Published:** 2023-08-12

**Authors:** Tugba Akinci D’Antonoli, Renato Cuocolo, Bettina Baessler, Daniel Pinto dos Santos

**Affiliations:** 1 Institute of Radiology and Nuclear Medicine, Cantonal Hospital Baselland, Liestal, Switzerland; 2 Department of Medicine, Surgery and Dentistry, University of Salerno, Baronissi, Italy; 3 Department of Diagnostic and Interventional Radiology, University Hospital Würzburg, Würzburg, Germany; 4 Department of Radiology, University Hospital of Cologne, Cologne, Germany; 5 Department of Radiology, University Hospital of Frankfurt, Frankfurt, Germany

**Keywords:** Multiomics, Radiomics, Artificial intelligence, Reproducibility of results

## Abstract

**Objectives:**

To investigate the model-, code-, and data-sharing practices in the current radiomics research landscape and to introduce a radiomics research database.

**Methods:**

A total of 1254 articles published between January 1, 2021, and December 31, 2022, in leading radiology journals (*European Radiology*, *European Journal of Radiology*, *Radiology*, *Radiology: Artificial Intelligence*, *Radiology: Cardiothoracic Imaging*, *Radiology: Imaging Cancer*) were retrospectively screened, and 257 original research articles were included in this study. The categorical variables were compared using Fisher’s exact tests or chi-square test and numerical variables using Student’s *t* test with relation to the year of publication.

**Results:**

Half of the articles (128 of 257) shared the model by either including the final model formula or reporting the coefficients of selected radiomics features. A total of 73 (28%) models were validated on an external independent dataset. Only 16 (6%) articles shared the data or used publicly available open datasets. Similarly, only 20 (7%) of the articles shared the code. A total of 7 (3%) articles both shared code and data. All collected data in this study is presented in a radiomics research database (*RadBase*) and could be accessed at https://github.com/EuSoMII/RadBase.

**Conclusion:**

According to the results of this study, the majority of published radiomics models were not technically reproducible since they shared neither model nor code and data. There is still room for improvement in carrying out reproducible and open research in the field of radiomics.

**Clinical relevance statement:**

To date, the reproducibility of radiomics research and open science practices within the radiomics research community are still very low. Ensuring reproducible radiomics research with model-, code-, and data-sharing practices will facilitate faster clinical translation.

**Key Points:**

*• There is a discrepancy between the number of published radiomics papers and the clinical implementation of these published radiomics models.*

*• The main obstacle to clinical implementation is the lack of model-, code-, and data-sharing practices.*

*• In order to translate radiomics research into clinical practice, the radiomics research community should adopt open science practices.*

## Introduction

Radiomics is an image analysis method that makes it possible to discover more than what can be seen with the naked eye, and it is a rapidly growing field of research [1]. Since its first appearance in the literature [2], thousands of articles have been published [3]. At the turn of a new decade of radiomics research, the clinical translation of radiomics models still lags far behind [3]. One of the main reasons for this chasm between research and clinical implementation is the lack of reproducibility. Shortage of clinical data- or radiomics model-sharing practices in the field further complicates the achievement of this goal [4]. This trend resembles the reproducibility crisis in the field of psychology, where independent researchers attempted to replicate results of previously published prominent articles, but succeeded only 30% of the time [5]. Open science practices [6, 7], i.e., data and code sharing, are important steps to overcome these challenges, as they enable more researchers to independently test proposed methods on an independent dataset.

With the development of checklists such as CheckList for EvaluAtion of Radiomics research (CLEAR) [8], the Checklist for Artificial Intelligence in Medical Imaging (CLAIM) [9], or Must AI Criteria 10 Checklist (MAIC-10) [10], code and data sharing in radiomics and artificial intelligence (AI) research is increasingly encouraged. Despite the fact that these practices are being promoted, they may not be necessarily sufficient for reproducibility, i.e., authors may only share “pseudocode” that is only a representation of the main code, or there may be errors in the code even though it is fully shared [11]. This trend can also be observed in AI research in radiology: According to a recent article, data were released in only 13% of AI papers, code was shared in only 34%, and only 33% of shared code was reproducible [12].

In the absence of a universally accepted definition of reproducibility, in this paper, we accept the earlier description of “research reproducibility” [13, 14]. Namely, research is *reproducible* if the same results are obtained when the same data, code, and method are used, and research is *replicable* if the same results are obtained when the same method and code are applied on an independent dataset. Considering these definitions, reproducibility is the minimum criterion for generalizable radiomics research [14].

In this paper, we have explored the practices of sharing model, code, and data in the current radiomics research landscape. Along with presenting our findings in this paper, we also introduce a large radiomics research database to facilitate the retrieval of radiomics models, code, and data if shared. We believe the radiomics research database will help researchers disseminate scientific knowledge and pave the way for clinical implementation, and we hope that this will encourage more researchers to share their model, code, and data. We will continue to expand the radiomics research database and call on interested researchers to help us in this endeavour.

## Material and methods

### Article selection and screening

A retrospective search using the following string was in the PubMed and Embase databases: (“radiomics”[Title/Abstract] OR “radiomic”[Title/Abstract]) AND (“Radiology”[Journal] OR “Radiology Artificial Intelligence”[Journal] OR “European Radiology”[Journal] OR “European Journal of Radiology”[Journal]) AND 2021/01/01:2021/12/31[Date—Publication].

**Fig. 1:**
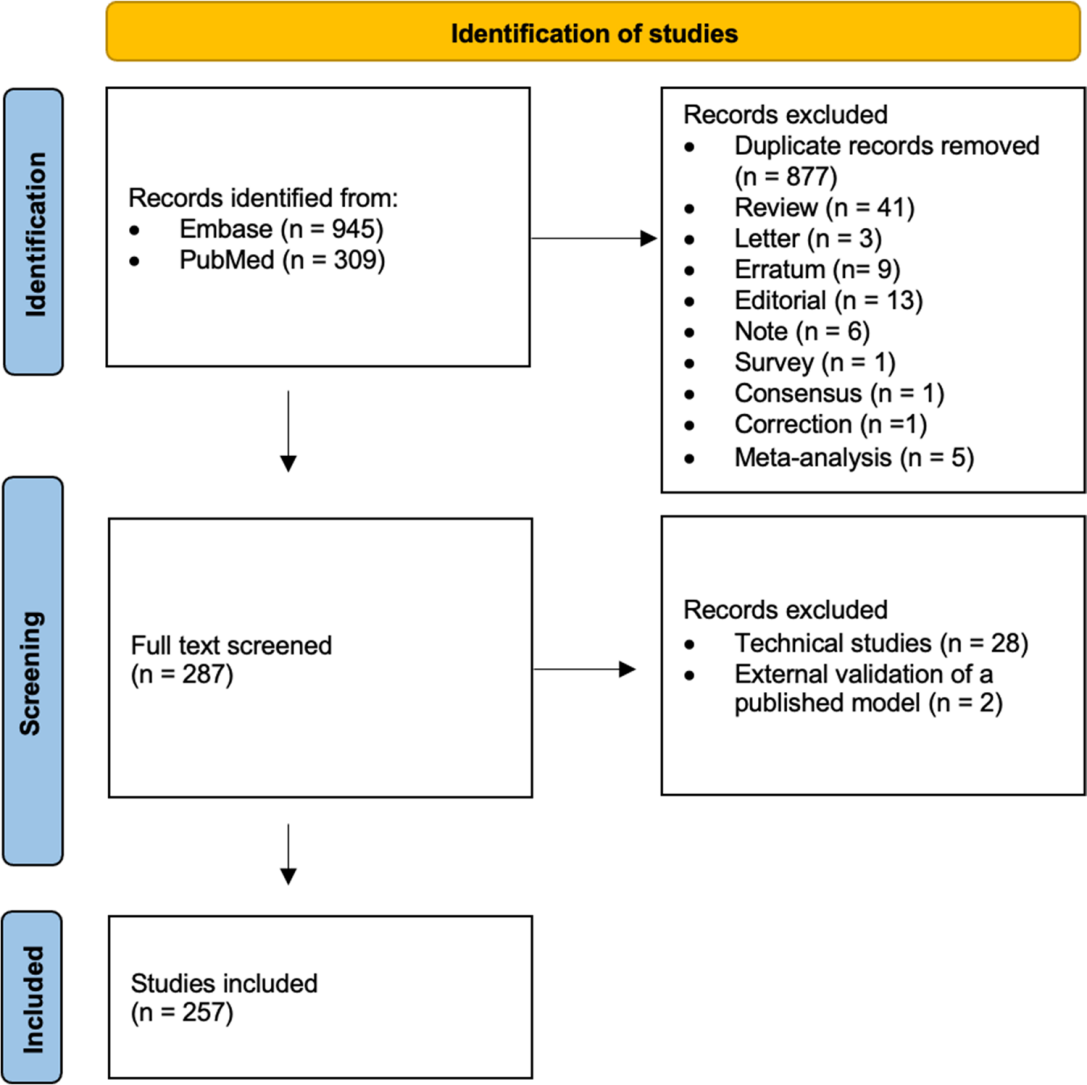
Study flow

All articles published between January 1, 2021, and December 31, 2022, were reviewed to identify the original research studies using “radiomics” or “radiomic” keywords in the title or abstract. We randomly selected leading first quarter (Q1) journals from Europe and the USA, namely *European Radiology*, *European Journal of Radiology*, *Radiology*, *Radiology: Artificial Intelligence*, *Radiology: Cardiothoracic Imaging*, and *Radiology: Imaging Cancer.* First, articles that were not original, i.e., systematic reviews, meta-analyses, literature reviews, editorials, letters, and corrections, were excluded by article type, title, and abstract screening. Second, technical studies testing robustness of features and external validation studies validating previously published models were also excluded by full-text screening (Fig. 1).

All studies were screened by four radiologists (T.A.D., 6 years of experience in research and radiology; D.P.d.S., 13 years of experience in research and radiology; R.C., 11 years of experience in research and radiology; B.B., 12 years of experience in research and radiology).

### Article characteristics

Four raters then extracted the following data from selected articles: article title, digital object identifier (DOI), journal title, first author, correspondence address, country, study design, main topic, subtopic, modality, model method, model shared or not, link to model, data shared or not, link to data, code shared or not, and link to code. The raters were also responsible for reviewing the code or data if shared. We explicitly only considered internal validation available if done on a hold-out set because that would be more useful in terms of clinical relevance. When a study used only cross-validation for internal validation, it was considered as a study that did not meet the criterion for internal validation, as no independent test data was available. If cross-validation is to be used alone, the entire pipeline must be nested, or a test set should be left out of the pipeline to obtain unbiased results; otherwise, the model will be overfitted and cannot be generalized [15, 16]. The studies that used a public dataset were considered to have shared the data. Disagreements between raters were resolved by consensus, if any.

### Statistical analysis

Descriptive statistics were used to summarize article characteristics as well as code- and data-sharing practices. A comparison of categorical variables was performed using the Fisher exact test or chi-square test and numerical variables using Student’s *t* test. Results were also compared between publication years 2021 and 2022. All analyses were performed with STATA program (IC version 15.1). The alpha value was set to 0.05.

## Results

### Article screening and characteristics

A total of 257 original research articles that describe the development and evaluation of a new radiomics model were included in this study (Fig. 1). Most of the included articles were published in *European Radiology* (76%; 195 of 257), followed by *European Journal of Radiology* (17%; 44 of 257). The total number of published articles increased from 106 in 2021 to 151 in 2022 (Table 1).

**Table 1:** Breakdown of article characteristics

Characteristics	*n* (%)
Publication year	
2021	106
2022	151
Journal	
* European Radiology*	195 (75.9%)
* European Journal of Radiology*	44 (17.1%)
* Radiology*	12 (4.7%)
* Radiology: Imaging Cancer*	4 (1.6%)
* Radiology: Artificial Intelligence*	2 (0.8%)
Topic	
Abdominal	52 (20.2%)
Chest	52 (20.2%)
Neuroradiology	43 (16.7%)
Genitourinary	36 (14.0%)
Head and neck	26 (10.1%)
Breast	21 (8.2%)
Musculoskeletal	15 (5.8%)
Cardiovascular	4 (1.6%)
Oncology	3 (1.2%)
Pediatric	3 (1.2%)
Chest/neuroradiology	1 (0.4%)
Chest/abdominal	1 (0.4%)
Modality	
MRI	110 (42.8%)
CT	109 (42.4%)
PET/CT	17 (6.6%)
US	15 (5.8%)
Mammography	3 (1.2%)
Angiography	1 (0.4%)
X-ray	1 (0.4%)
MRI, CT	1 (0.4%)
Study design	
Retrospective	248 (96.5%)
Prospective	8 (3.11%)
Model utility	
Classification	144 (56.03%)
Prognostication	79 (30.74%)
Detection	30 (11.67%)
Classification, prognostication	4 (1.55%)
Model type	
LASSO/LR	139 (54.4%)
ML classifiers*	64 (24.9%)
RF**	20 (7.7%)
SVM***	20 (7.7%)
DL	11 (4.2%)
Not mentioned	3 (1.1%)

Abbreviations: *LASSO* least absolute shrinkage and selection operator, *LR* logistic regression, *RF* random forest, *SVM* support vector machine, *DL* deep learning, *ML* machine learning

^*^In most cases, multiple ML modeling methods were combined. Some of the methods are listed here: linear classifier, K-nearest neighbor, passive-aggressive classifier, perceptron, ridge classifier, AdaBoost, Naïve-Bayes, ElasticNet, Gradient Boosting Decision Tree, AutoML Ensemble

^**^The number of papers only used the random forest method for modelling, but not as a part of combined ML classifiers

^***^The number of papers only used the support vector machine method for modelling, but not as a part of combined ML classifiers

**Fig. 2:**
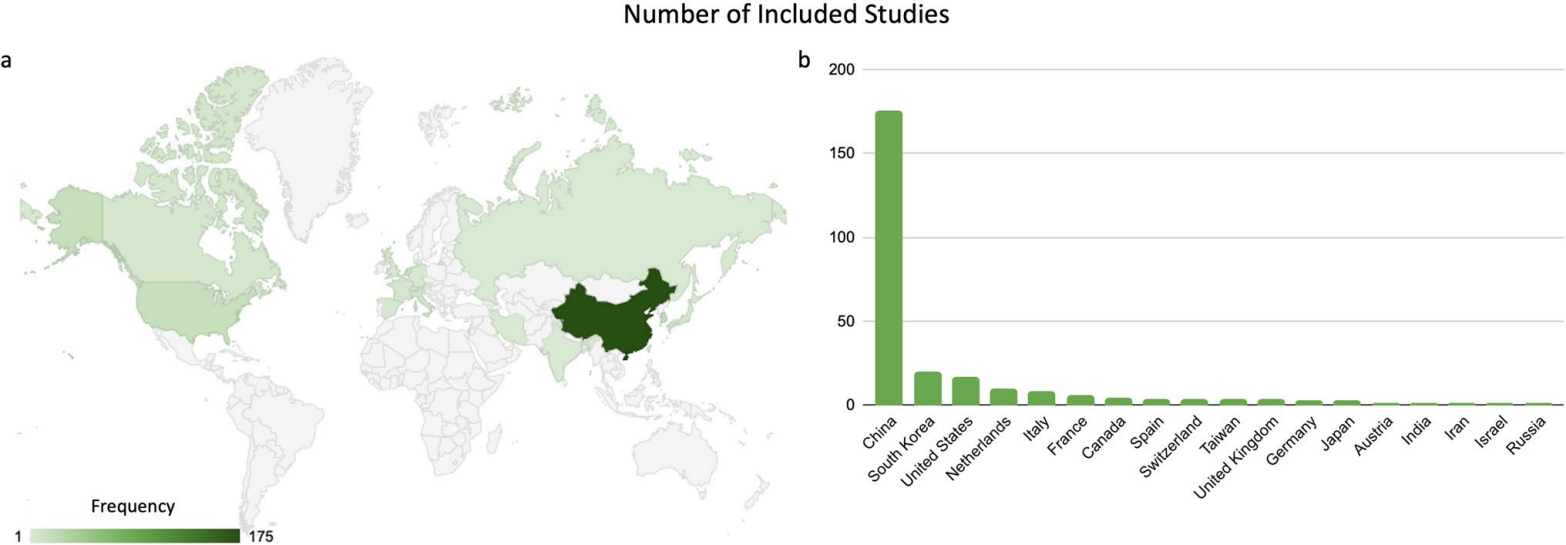
**a** Geo chart and (**b**) bar graph showing the number of published research papers in the field of radiomics that were included this study, breakdown by country. The darker the green, the higher the number of published papers, and gray means that there are no publications from that country in the geo chart. For example, in 2021–2022, there were 175 articles published from China, while there was only 1 article published from India. Note that the Mercator projection is used for the geo chart, which may not be representative of the actual surface area

Most of the articles were from China (68%; 175 of 257), followed by the Republic of Korea (7%; 19 of 257) and the USA (6%; 16 of 257) (Fig. 2). Most of the included articles focused on chest (20%; 52 of 257) and abdominal radiology (20%; 52 of 257). The main modalities used in the included studies were CT (42%; 109 of 257) and MRI (42%; 110 of 257). The study design was mainly retrospective (96%; 249 of 257). Only 4% (8 of 257) of the included studies were prospective, and of the 8 studies, only 1 was a randomized controlled trial. Interestingly, we found that one retrospective study was incorrectly reported as a prospective study because the model was trained on a retrospective dataset but validated on a prospective dataset, i.e., temporal validation. In most cases, the model was developed for classification tasks (56%; 144 of 257) and the second most commonly for prognostication tasks (30%; 79 of 257) (Table 1).

### Open science practices

To evaluate the reproducibility of radiomics research in this study, we adapted a previously proposed reproducibility classification framework [17]: statistical reproducibility, i.e., the validated model and details of modeling methods are shared; conceptual reproducibility, i.e., external validation is performed; and technical reproducibility, i.e., code and/or data are available. All results are shown in Tables 2 and 3.

**Table 2:** The number of articles following open science practices by publication year

	2021 (*n* = 106)	2022 (*n* = 151)	*p*
Model shared	58 (54.7%)	70 (46.4%)	0.19
Internal validation			
Hold-out set	78 (73.6%)	104 (68.9%)	0.41
None			0.02
Bootstrap	1 (0.9%)	0 (0.0%)	
Cross-validation	1 (0.9%)	15 (9.9%)	
None	26 (24.5%)	32 (21.2%)	
External validation	23 (21.7%)	50 (33.1%)	0.05
Open dataset	7 (6.6%)	9 (6.0%)	0.83
Open code	4 (3.8%)	16 (10%)	0.06

Data in parentheses are within column percentages

**Table 3:** The number of articles following open science practices breakdown by journal

Journal	Model shared (*n* = 128)	Internal validation (*n* = 182)	Cross-validation (*n* = 16)	Bootstrap (*n* = 1)	External validation (*n* = 73)	Open dataset (*n* = 16)	Open code (*n* = 20)
*European Radiology* (*n* = 195)	102 (52%)	139 (71%)	13 (7%)	1 (0.5%)	54 (28%)	10 (5%)	7 (3%)
*European Journal of Radiology* (*n* = 44)	20 (45%)	33 (75%)	0 (0.0%)	0 (0.0%)	10 (23%)	2 (4%)	5 (11%)
*Radiology* (*n* = 12)	6 (50%)	7 (58%)	1 (8%)	0 (0.0%)	7 (58%)	2 (17%)	6 (50%)
*Radiology: Imaging Cancer* (*n* = 4)	0 (0.0%)	2 (50%)	1 (25%)	0 (0.0%)	1 (25%)	1 (25%)	0 (0.0%)
*Radiology: Artificial Intelligence* (*n* = 2)	0 (0.0%)	1 (50%)	1 (50%)	0 (0.0%)	1 (50%)	1 (50%)	2 (100%)

Data in parenthesis are within row percentages

#### Statistical reproducibility

Half of the articles (128 of 257) included either the final model formula or selected radiomics features along with their coefficients. We accepted that either approach was sufficient to meet the model-sharing requirements, and we observed no difference between the years 2021 and 2022 (*p* = 0.19). Of the 257 articles, two indicated that the model was shared in the supplement; however, upon assessment of all available supplementary material, no model was found; thus, for the purpose of our analysis, these were included as articles that did not share the model.

The most commonly used modelling method was least absolute shrinkage and selection operator or logistic regression (LASSO/LR; 54%; 139 of 257). Moreover, 72% (100 of 139) of the LASSO/LR models were shared. The second most common modelling method was using a combination of several machine learning (ML) classifiers, e.g., K-nearest neighbour (KNN), Naïve-Bayes, ElasticNet, Gradient Boosting Decision Tree (XGBoost), and AdaBoost; 64 of 257 (25%) and 15 of 64 (23%) ML models were shared. Most commonly sole used machine learning classifiers were random forest (RL; 7%; 20 of 257) and support vector machine (SVM; 7%; 20 of 257) and 4 of 20 (20%) RF and 7 of 20 (35%) of SVM models were shared. Only 4% of the modelling methods was deep learning (DL; 11 of 257) and only 2 of 11 (18%) DL models were shared. Interestingly, in 3 of the 257 articles, the researchers indicated no modelling method at all (1%).

Most of the papers validated the model on a hold-out set internally (71%; 182 of 257), and this practice did not change between the years 2021 and 2022 (*p* = 0.41). We noted some researchers performed only cross-validation (6%; 16 of 257) rather than validating their model on a hold-out set, and hence, as detailed above for the purpose of this manuscript, this approach was assessed as not having internal validation. The number of papers using only cross-validation increased from 1 to 15 from 2021 to 2022 (*p* = 0.018).

#### Conceptual reproducibility

Only 28% (73 of 257) of the models were externally validated on an independent dataset, and the number of externally validated models has increased from 23 to 50 within one year (*p* = 0.046).

#### Technical reproducibility

Only 6% (16 of 257) of the included articles shared the data or used publicly available open datasets. Similarly, only 7% (20 of 257) of the articles included a link to the code used for data analysis. Of the 20 articles with a link to the code, one included only pseudocode. Only 3% (7 of 257) of articles both shared code and data at the same time.

Table 3 shows a breakdown of the number of articles sharing code and data by the journal.

### Radiomics research database (RadBase)

As a first step, the collected data in this article has been made available as a comma-separated value (CSV) file, but we hope to be able to add and refine more data in the near future continuously. We have chosen to make the database available on GitHub as this allows interaction, e.g., if a researcher wants to update fields pertaining to their study or if others want to contribute new entries, this can be done via issues or pull requests. In the future, authors and journals could also proactively register their studies in the database with a link to the code before or after the publication.

For this first version of the database, we have provided instructions for accessing the CSV file directly so that researchers can create scripts to notify them of updates.

Our initial database, *RadBase*, containing all information from the articles we included in this study can be accessed at https://github.com/EuSoMII/RadBase.

## Discussion

In this study, we found that most radiomics studies did not share code or data; in other words, most radiomics models were not reproducible. To our surprise, the authors tend to share their validated model with an extensive model-building methodology; however, only around 30% of the models were conceptually reproducible as they were validated on an external dataset. In this study, we also introduced a searchable radiomics research database (*RadBase*). To our knowledge, there has been only one such initiative within the AI research community: the *Papers with Code* website shares papers that contain links to code to promote code sharing [18]. With the proposed radiomics database, we aim to promote all facets of reproducible radiomics research and achieve broader acceptance within the radiomics community.

Despite the growing number of guidelines encouraging open science practices in radiomics research, our results show that the radiomics research community still lags far behind. In addition, some researchers find a way to circumvent checklists and guidelines by intentionally or unintentionally sharing broken links, empty GitHub repositories, incomplete code, or data that never allow published methods to be truly reproduced [12]. In this study, we also observed practices such as misleading statements, e.g., reporting that the model is in the supplement but it was not [19, 20], and sharing only pseudocode [21] that does not allow to reproduce proposed model. To avoid these issues, not only code or data sharing but also reproducible code sharing is encouraged by major artificial intelligence congresses such as Neural Information Processing Systems (NeuroIPS) with the Machine Learning Reproducibility checklists [22].

At the turn of a decade, we should embark on reproducible radiomics research if we do not want to spend another decade waiting for clinical implementation. We should also ensure that decades later our methodology can withstand the challenges that come with the ever-changing technologies. The results of a recent initiative to determine whether the code that researchers wrote 10 years ago still works [23] can serve as a cautionary tale. While some of the researchers were able to get 10-year-old code to work, even when the data had to be transferred from a floppy disk to the cloud, the majority of them (80%) did not work [24]. Unfortunately, our results over the last 2 years point in the same direction—most of the radiomics models were technically not reproducible. In an ever-growing field of radiomics research, as we adopt open science practices, we should also make sure that our methods persist over time.

Similar to a previous study [12], we observed that authors who were required to make a data- and code-sharing statement were more likely to share their data and code openly. Promoting reproducibility thorough journal standards, checklists, and badges could enable the widespread adoption of open science practices [25]. Moreover, this could be as simple as requesting a statement to do so [6]. Journals could adopt the proposed model of standards for reproducibility to reward these practices accordingly on a sliding scale [6, 25]. Evidently, the model and code have to be submitted with the manuscript to ensure reproducibility. However, an exception may be made if the authors can demonstrate that they wish to protect their intellectual property for a while, but if they fail to do so within a reasonable time frame, the manuscript should be withdrawn or modified. Clearly, patient privacy issues could hinder data sharing in healthcare, but this could be easily overcome by sharing data through databases with strict data use agreements.

Our study had some limitations. First, we only included a selection of articles from leading journals that were recently published. Therefore, our results might not be generalizable to the rest of the radiomics literature. Even if we were able to show that publications in some of the highest-ranked journals from Europe and the USA only rarely followed open-science practices, this result could be different in other journals from different parts of the word (e.g., the journals from the Asia–Pacific region). Secondly, we did not technically control if the shared model is reproducible or not, but we encourage researchers to join our efforts and use our database for the planning and execution of validation studies. Finally, although we did not extract data on methodological details of the entire study pipeline in this study, e.g., postprocessing methods, feature extraction techniques, feature normalization, or harmonization methods, these details could also affect the reproducibility of the study and should be properly reported. Therefore, we encourage researchers to share data not only concerning statistical, conceptual, or technical reproducibility, such as we collected in this study, but also the data on the whole pipeline in our database.

In conclusion, there is room for improvement regarding reproducibility in radiomics research. We urge researchers to adopt open science practices to ensure reproducible radiomics research now and in the future.

## Abbreviations

AI: Artificial intelligence
CLAIM: Checklist for Artificial Intelligence in Medical Imaging
CLEAR: CheckList for EvaluAtion of Radiomics research
CSV: Comma-separated value
DOI: Digital object identifier
KNN: K-nearest neighbor
LASSO/LR: Least absolute shrinkage and selection operator or logistic regression
MAIC-10: Must AI Criteria 10 Checklist
ML: Machine learning
NeuroIPS: Neural Information Processing Systems
Q1: First quarter
RF: Random forest
SVM: Support vector machine
XGBoost: Gradient Boosting Decision Tree

## Declarations

### Guarantor

The scientific guarantor of this publication is Daniel Pinto dos Santos.

### Statistics and biometry

No complex statistical methods were necessary for this paper.

### Methodology

• retrospective

• observational

• multicenter study
